# Realization of a PEDOT:PSS/Graphene Oxide On-Chip Pseudo-Reference Electrode for Integrated ISFETs

**DOI:** 10.3390/s22082999

**Published:** 2022-04-14

**Authors:** Marcel Tintelott, Tom Kremers, Sven Ingebrandt, Vivek Pachauri, Xuan Thang Vu

**Affiliations:** Institute of Materials in Electrical Engineering 1, RWTH Aachen University, Sommerfeldstr. 24, 52074 Aachen, Germany; tintelott@iwe1.rwth-aachen.de (M.T.); tom.kremers@rwth-aachen.de (T.K.); ingebrandt@iwe1.rwth-aachen.de (S.I.); pachauri@iwe1.rwth-aachen.de (V.P.)

**Keywords:** PEDOT:PSS, 2D materials, biosensor, stability, gate electrode, diffusion barrier

## Abstract

A stable reference electrode (RE) plays a crucial role in the performance of an ion-sensitive field-effect transistor (ISFET) for bio/chemical sensing applications. There is a strong demand for the miniaturization of the RE for integrated sensor systems such as lab-on-a-chip (LoC) or point-of-care (PoC) applications. Out of several approaches presented so far to integrate an on-chip electrode, there exist critical limitations such as the effect of analyte composition on the electrode potential and drifts during the measurements. In this paper, we present a micro-scale solid-state pseudo-reference electrode (pRE) based on poly(3,4-ethylene dioxythiophene): poly(styrene sulfonic acid) (PEDOT:PSS) coated with graphene oxide (GO) to deploy with an ion-sensitive field-effect transistor (ISFET)-based sensor platform. The PEDOT:PSS was electropolymerized from its monomer on a micro size gold (Au) electrode and, subsequently, a thin GO layer was deposited on top. The stability of the electrical potential and the cross-sensitivity to the ionic strength of the electrolyte were investigated. The presented pRE exhibits a highly stable open circuit potential (OCP) for up to 10 h with a minimal drift of ~0.65 mV/h and low cross-sensitivity to the ionic strength of the electrolyte. pH measurements were performed using silicon nanowire field-effect transistors (SiNW-FETs), using the developed pRE to ensure good gating performance of electrolyte-gated FETs. The impact of ionic strength was investigated by measuring the transfer characteristic of a SiNW-FET in two electrolytes with different ionic strengths (1 mM and 100 mM) but the same pH. The performance of the PEDOT:PSS/GO electrode is similar to a commercial electrochemical Ag/AgCl reference electrode.

## 1. Introduction

In recent years, field-effect transistors (FETs) have become one of the most promising sensor platforms for the electronic detection of biomolecules. Besides the well-known ion-sensitive field-effect transistor (ISFET) [1] and its nanoscale counterpart, the silicon nanowire field-effect transistor (SiNW-FET) [2], several new materials, such as graphene [3], reduced graphene oxide [4,5], carbon nanotubes [6], poly(3,4-ethylene dioxythiophene): poly(styrene sulfonic acid) (PEDOT:PSS) [7], or zinc oxide nanowires [8], have been investigated for FET-based (bio)sensing applications. Even though the working principle of these devices is based on different physical phenomena, a stable reference electrode (RE) is mandatory for reliable and reproducible measurements. Commonly, bulky and often fragile electrochemical silver/silver chloride (Ag/AgCl) REs are used to provide a stable gate potential to operate an ISFET for the detection of biomolecules or other analytes. An electrochemical Ag/AgCl RE requires a chloride solution at a given concentration (e.g., 3 M KCl), surrounding the Ag/AgCl wire in a container, and an ion-conductive membrane allowing electrical contact between the electrode and the electrolyte solution. Due to the need for a membrane and an electrolyte solution, the miniaturization of an electrochemical RE remains challenging [9,10,11].

With the advancement of nanoscale fabrication methods, the transduction area of FET-based sensors has shrunk down to a few tens of nanometers [12,13]. While the sensors and microfluidic systems are becoming smaller, the average sizes of the mandatory RE have remained virtually the same [9]. Apart from the miniaturization on its own, miniaturized and integrated micro-scale REs are expected to eliminate current limitations, such as the use of large sample sizes, microfluidic integration towards Lab-on-a-Chip (LoC) systems, or better portability of (bio)sensor systems [12,14]. An on-chip micro-scale RE should ideally be an entirely solid-state building-block to prevent electrolyte leakages and exert a potential independent of the electrolyte or analyte [9]. Several concepts of on-chip pREs exist, such as Ag/AgCl redox systems or those utilizing the catalytic properties of platinum or iridium oxide. Such pREs, however, either exhibit low potential stability or show high pH or ionic cross-sensitivity [10,15,16,17].

Conductive polymers have been used in various applications, especially for biological and biochemical sensing applications. PEDOT:PSS is one of the most studied and promising conductive polymers. It is a polymer–polyelectrolyte complex that offers both ion and electron conductivity with semiconducting and redox-active charge conduction properties [18,19], making it a popular material, often used in organic electrochemical transistors (OECTs) [20], light-emitting diodes (LEDs) [21], biohybrid synapse [22], neural probes [23], ion-selective electrodes [24], and ion-pumps [25]. As a material, PEDOT:PSS has shown the ability of coupling between ionic and electronic species [26,27]. The electronic conduction of the π-conjugated PEDOT:PSS is based on weakly bound electrons that can move along a molecule through delocalized π-orbitals and between different molecules if a sufficient π-π overlap is present. However, delocalization and overlap are limited by the structural disorder in the material. In this case, thermally activated hops can describe the nature of the electrical conductivity of PEDOT:PSS. Different models, such as ion hopping, solvated/vehicle, and Grotthuss mechanisms, can describe the ionic current [26,27]. The ionic-electronic interaction is based on either electrostatic ion-electron coupling or direct electron transfer [26,27]. In addition to its electrical properties, the selective coating of PEDOT:PSS (e.g., using electropolymerization) onto metallic substrates allows rapid, low-cost, and high throughput fabrication. Conductive polymers have been used as pREs in various applications, such as electrochemical impedance spectroscopy (EIS) [28,29], ISFETs [30], or OECTs [31]. Due to its ion conductivity, a high cross-sensitivity to the ion concentration of the electrolyte can be expected [32]. Therefore, an ion diffusion barrier that does not degrade the electrode performance but eliminates the cross-sensitivity to ions would be of great interest to increase the stability of polymeric pREs. Here, graphene and graphene derivatives (e.g., graphene oxide (GO)) have proven to be excellent materials to prevent diffusion due to their pinhole-free layers and close interlayer distance packing [33,34,35,36]. However, the large-scale deposition of graphene is still challenging [37]. GO, by contrast, allows solution-based processing, which is compatible with standard cleanroom processes and, therefore, enables high throughput and low-cost fabrication [4,38].

In this article, we present a solid-state pRE based on PEDOT:PSS coated with GO as an ion diffusion barrier, which exhibited a highly stable long-term potential and reduced cross-sensitivity to the ionic strength of the electrolyte. Furthermore, we used SiNW-FETs to perform well-known pH experiments. Here, a SiNW-FET gated with the presented pRE exhibited a similar performance as the ones gated with a commercial electrochemical Ag/AgCl RE. To evaluate the cross-sensitivity to changes in ion concentration, measurements were carried out at the same pH but with different ion concentrations. A GO-coated PEDOT:PSS electrode showed a significantly reduced cross-sensitivity to the ionic strength of the electrolyte compared to a bare PEDOT:PSS electrode. Furthermore, we could show that the quality of the GO layer on top of the polymeric electrode has a huge impact on the reliability of SiNW-FETs. The performance of the PEDOT:PSS/GO electrode is similar to a commercial electrochemical Ag/AgCl reference electrode.

## 2. Materials and Methods

### 2.1. Materials

Phosphate-buffered saline (PBS) was prepared by dissolving pH buffer capsules (Sigma-Aldrich Chemie GmbH, Taufkirchen, Germany) in deionized (DI) water. Phosphate buffer solutions (pH 7, 100 mM) were prepared by dissolving sodium phosphate dibasic dihydrate and sodium phosphate monobasic monohydrate (Sigma-Aldrich Chemie GmbH, Taufkirchen, Germany) in DI water. The 1 mM phosphate buffer was prepared by the dilution of a 100 mM phosphate buffer using DI water. The pH was measured using a HI5522 pH-meter (Hanna Instruments Deutschland GmbH, Vöhringen, Germany). A leak-free Ag/AgCl double junction RE (DRIREF-2SH, World Precision Instruments, Sarasota, FL, USA) was used as a reference. The GO solution was synthesized using low-temperature exfoliation as described before [39]. The chemical exfoliation was performed using the improved Hummers method.

### 2.2. Electrode Fabrication

The deposition of PEDOT:PSS was carried out by the electropolymerization of a mixture of 3,4-ethylene dioxythiophene (EDOT) and PSS. Both solutions were dissolved in ultra-pure DI water with a concentration of 20 mM, respectively. An EG&G Model 283 Potentiostat/Galvanostat (Princeton Applied Research, Oak Ridge, TN, USA) was used for depositions of PEDOT:PSS on Ti/Au microelectrodes. Defined charge depositions were carried out potentiostatically at 1 V vs. Ag/AgCl. The potentiostat terminated the deposition when a predetermined total charge was transferred. This deposition method was used to obtain reproducible film characteristics for depositions using the same parameters. Assuming that the ohmic current between the counter electrode (CE) and the working electrode (WE) was negligible in contrast to the current flow caused by the electropolymerization at the electrode, multiple depositions with the same defined charge and electrode area should lead to the same amount of monomers reacting and therefore to the same film thickness. The GO coating of the electrodes was performed using the drop-casting technique. An amount of 10 mL of the GO solution obtained from an exfoliation process developed earlier [4] was drop-casted on the electrode area and dried for 5 min at 50 °C. Accordingly, the choice of charge is dependent on the electrode size and the desired film thickness. Table 1 provides an overview of the four different pREs used in this study.

### 2.3. SiNW-FET Fabrication

The SiNW-FETs were fabricated based on the “top-down” approach using a mix & match process involving electron beam lithography (EBL) and photolithography on a 4-inch wafer scale. Briefly, a silicon-on-insulator (SOI) wafer (Soitec, Bernin, France) with a 70 nm top silicon layer and 145 nm buried-oxide (BOX) was thinned down to ~50 nm by thermal oxidation to define the resulting height of the nanowires. The resulting oxide was used as a hard mask for wet chemical patterning of the top silicon layer [40]. The nanowires and the drain and source regions were defined by EBL and optical lithography using mix & match resist (AR-N7520.11 new, Allresist GmbH, Strausberg, Germany). After the resist development, a CHF_3_ dry etching process was carried out to selectively transfer the resist structures onto the silicon oxide hard mask. Tetramethylammonium hydroxide (TMAH) solution (25%) was used to etch the top silicon layer, selectively [41]. An ion implantation process was carried out with a dose of 5 × 10^15^ atoms/cm², implantation energy of 8 keV, and a 7° tilt to form the drain and source regions. A combination of dry oxidation and silicon oxide deposition by high-quality plasma-enhanced chemical vapor deposition (PECVD) was used for the passivation of the drain and source feed lines [42]. The passivation was then etched away on the gate area and the drain and source contact area. A high-quality (dry oxidation process) silicon oxide (~7 nm) was grown on the SiNW gate areas acting as a gate dielectric layer. On-chip temperature sensors and pREs were fabricated using optical lithography combined with a layer stack of chromium, platinum, and titanium lift-off process. A low-temperature oxidation process was carried out to oxidize the top layer of titanium to passivate the temperature sensors and the contact line of the electrode. The TiO_2_ on top of the electrode was removed by optical lithography and wet etching in buffered HF (BOE 71). A layer stack of aluminum (150 nm), titanium (10 nm), and Au (100 nm) was deposited by electron beam evaporation on the source and drain contact areas to form reliable ohmic contacts with the SiNWs. Before the metal evaporation, an HF-dip was performed to remove native SiO_2_ from the source and drain contact pads. Finally, the wafer was annealed at 350 °C for 10 min in forming gas (N_2_/H_2_) to create ohmic contacts. Further descriptions of the SiNW-FET fabrication process can be found in a previous publication [43].

### 2.4. Impedance Measurements

The PEDOT:PSS and PEDOT:PSS/GO films were initially investigated using EIS. The electrode under test was connected to the WE while an Ag/AgCl pellet served as the CE, and an electrochemical Ag/AgCl RE was connected to the RE port. A Novocontrol Technologies Alpha-A High-Performance Frequency Analyzer (Novocontrol Technologies GmbH & Co. KG, Montabaur, Germany) was used to measure the impedance spectra. The spectra were obtained in the frequency range between 0.1 Hz and 1 MHz, with an applied voltage amplitude of 10 mV in 1× PBS (pH 7.4) as the electrolyte.

### 2.5. OCP Measurements

Open Circuit Potential (OCP) was recorded for different electrodes in phosphate buffer solution (1 mM, pH 7) using a Potentiostat/Galvanostat Model 283 (EG&G Instruments, Princeton Applied Research, Oak Ridge, TN, USA). A 2-electrode setup was used to measure the OCP of the electrode under test versus a leak-free Ag/AgCl double junction RE (DRIREF-2SH, World Precision Instruments, Inc., Sarasota, FL, USA).

### 2.6. Electrical Measurements

The characterization of the SiNW-FETs was performed using a Keithley 4200A-SCS (Keithley Instruments, Solon, OH, USA). A drain-source voltage of –0.1 V was applied between the drain and source terminals of the SiNW-FET. The gate voltage was applied to the RE. In general, reported gate potentials refer to the potential, which is applied to the RE, and not the effective voltage at the transistor gate. Characterizations were carried out with an Ag/AgCl electrode, GO-coated Au electrodes (pRE 1), PEDOT:PSS-coated Au electrodes (pRE 2), and PEDOT:PSS/GO-coated Au electrodes (pRE 3 and pRE 4). The threshold voltage has been extracted using the transconductance extrapolation method (see Figure S1).

## 3. Results and Discussion

### 3.1. Electrode Preparation and Characterization

**Figure 1:**
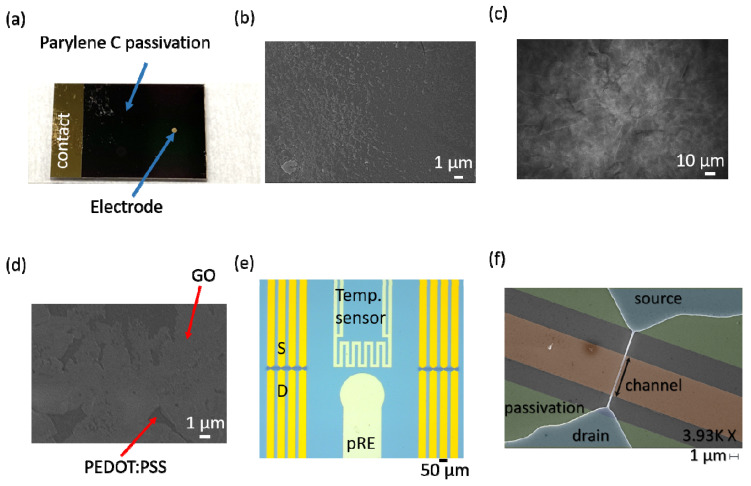
Photograph of the used electrode test structure (**a**). An image of a PEDOT:PSS electrode surface (**b**). SEM image of a PEDOT:PSS electrode coated with GO (**c**). High-resolution image of a GO-coated PEDOT:PSS electrode showing micro holes in the GO film (**d**) Microscopy image showing the SiNW arrays, an integrated temperature sensor, and an on-chip pRE (**e**). SEM image of a single SiNW-FET (**f**).

A 1 cm × 1.5 cm chip with a circularly patterned Au electrode, as shown in Figure 1a, was used to study the RE materials. The electrode had a diameter of 500 µm and thus an area of 0.169 mm². The fabrication involved the sputter deposition of a 30 nm thick titanium adhesion layer, 220 nm Au, and 50 nm titanium as a protective layer. Afterward, the chip surface was coated with a 3.5 µm thick Parylene C layer. A standard photolithography process was followed by a dry etching process to define the electrode and contact pad areas. This electrode design was chosen to match the electrode design of the fabricated SiNW-FET chip used in this work. The 1 cm × 1 cm SiNW-FET chip consisted of 16 individually addressable SiNW-FETs (~120 nm top width and 6 µm length (compare Figure 1f), monolithically integrated temperature sensors, and on-chip electrodes (Figure 1e).

The PEDOT:PSS was deposited on the designed electrode by electropolymerization of EDOT and PSS in DI water. A former study showed that our charge terminated deposition (combining a surface cleaning step and the actual electropolymerization process) results in a highly reproducible electrode coatings [29]. Before the electropolymerization process, the top Ti layer was etched using ammonium hydroxide-hydrogen peroxide solution to obtain a clean Au surface [44]. A charge terminated electropolymerization process was performed to coat the electrode surface with PEDOT:PSS. Scanning electron microscope (SEM) images were taken after each fabrication step to evaluate the respective process (Figure 1b–d). As shown in Figure 1b, the electropolymerization process resulted in a continuous PEDOT:PSS film. Figure 1c shows an SEM image of a PEDOT:PSS electrode coated with GO. The drop-casting of GO resulted in a continuous coating of the electrode surface (Figure 1c); however, several micro holes in the GO film were observed (Figure 1d).

**Figure 2:**
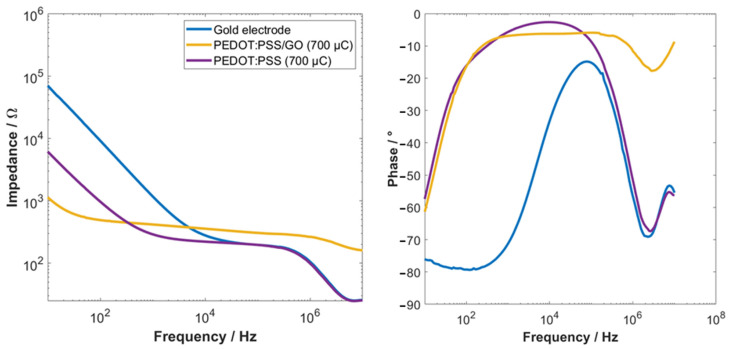
Bode plots of electrochemical impedance spectra (**left**) and phase (**right**) of an Au electrode, a PEDOT:PSS-coated Au electrode, and a GO-coated PEDOT:PSS electrode.

After each deposition, EIS measurements were performed on samples with different PEDOT:PSS deposition charges in PBS (pH 7.4) to determine the optimal coating parameters. As shown in Figure S2 of the supplementary material, a bare Au electrode exhibited the highest impedance compared to all electrodes coated with PEDOT:PSS. A higher termination charge for the electropolymerization of PEDOT:PSS resulted in a lower impedance, as shown in Figure S2, for termination charges of 10 µC, 1000 µC, and 10,000 µC, respectively. This test was performed to evaluate the electrode performance in dependency on the deposition charge. Even though the electrode coated with a deposition charge of 10,000 µC exhibited the lowest electrode impedance, this high deposition charge is not suitable for pREs. Firstly, the deposition of PEDOT:PSS took several hours, which was not beneficial for high throughput production. Secondly, such a lengthy deposition could induce significant variations in the electrode performance due to current flows, which do not originate from the electropolymerization process itself. The electropolymerization process could be accelerated by using a larger counter electrode. However, using a larger counter electrode resulted in the delamination of the PEDOT:PSS film (see Figure S3). Therefore, electrodes coated with a termination charge of 10 µC and 700 µC were identified for further investigations. Figure 2 shows the electrical impedance spectra of the electrode before and after the deposition of PEDOT:PSS and PEDOT:PSS coated with GO. Here, it can be seen that the deposition of PEDOT:PSS reduced the electrode impedance compared to a bare Au electrode as described before. An additional coating with GO further reduced the electrode impedance in the capacitive regime and slightly increased the impedance in the resistive regime. Furthermore, the percentage impedance change between 10 Hz and 100 kHz has been investigated. Here, a bare Au electrode exhibited an impedance change of ~36,000%, a PEDOT:PSS-coated electrode a change of ~3000%, and a PEDOT:PSS/GO electrode an impedance change of only 272%.

**Figure 3:**
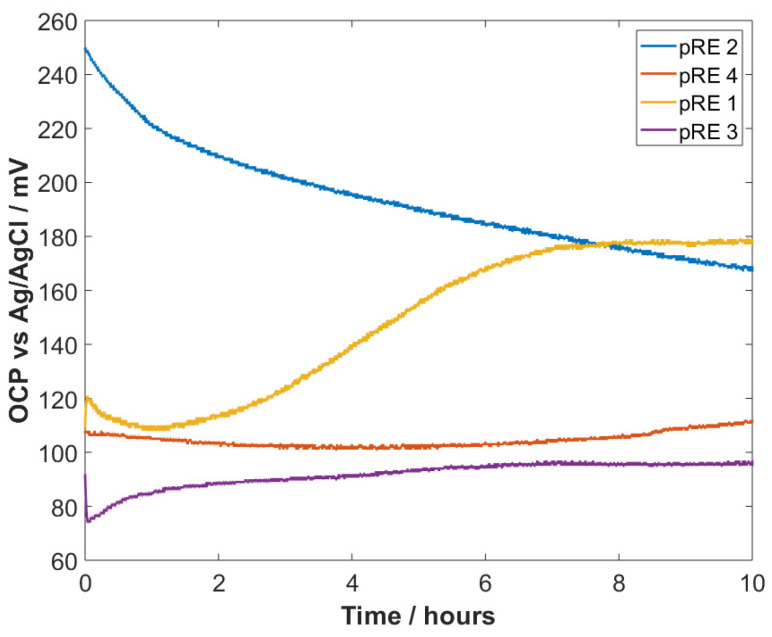
OCP measurements of four different electrodes against an electrochemical Ag/AgCl RE. pRE 4 exhibits the lowest drift, while the other electrodes exhibit an unstable OCP, especially within the first hour.

PEDOT:PSS-coated electrodes (termination charge of 10 µC and 700 µC) were coated with GO, using the drop-casting technique. To evaluate the potential stability of these electrodes, OCP measurements were performed in phosphate buffer (pH 7, 1 mM ion concentration). The measurements were performed in a 3D-printed fluidic chamber with insertion slots for the electrode under test and the RE (see Figure S4). The obtained OCPs of four different types of electrodes (PEDOT:PSS (pRE 2), an Au electrode coated with GO (pRE 1), and PEDOT:PSS coated with GO (pRE 3 + pRE 4)), which was recorded for 10 h, are presented in Figure 3. As shown in Figure 3, pRE 2 and pRE 1 exhibited an unstable OCP throughout the measurement with a significant drift. pRE 2 showed a high drifting rate for the first hour and a lower but continuous drifting rate with an overall OCP change of approximately 70 mV. Figure S9 shows additional transient OCP measurements for ~50 min and around 20 h, proving the drifting behavior of PEDOT:PSS-based electrodes. pRE 1 exhibited an OCP change of 10 mV within the first few minutes and remained unstable within the next 7 h, with a total drift of approximately 70 mV. pRE 3 showed an unstable OCP for the first hour and exhibited a lower drifting rate for the next 9 h. Superior results were observed for pRE 4. This electrode exhibited a minimal change in its OCP during the 10 h recording and did not show any significant OCP change in the first minutes after the immersion into the electrolyte. An inset of the first ~2 h can be found in the supporting information (Figure S5), which elucidates the differences in the OCP stability during the first minutes. Three additional OCP recordings for the pRE 4 are shown in the supporting information (Figure S6), proving the stable OCP for short-term measurements (20 min) and long-term measurements (3 h). Furthermore, two additional measurements are shown in the supporting information. Figure S7 shows an OCP recording over 3 days. Here, the electrode (pRE 4) exhibited a stable potential with a minimal drift for 10 h and a significant drift afterwards. As shown in Figure S8, the pRE 4 had the potential for minimal drifting. Here, the electrode exhibited a drift of 0.65 mV/h over the first 10 h and a slightly higher drift for the next 4.5 h. Table 2 shows the change in OCP at different times during the long-term recording shown in Figure 3. The percentage change in the OCP of the different pREs shows that pRE 1 and pRE 3 exhibited a highly unstable behavior within the first minutes. Furthermore, pRE 1 and pRE 2 showed a significant (larger than 39% within 10 h) OCP change. Compared to the starting point, the change in OCP of pRE 4 was always less than 10%.

**Figure 4:**
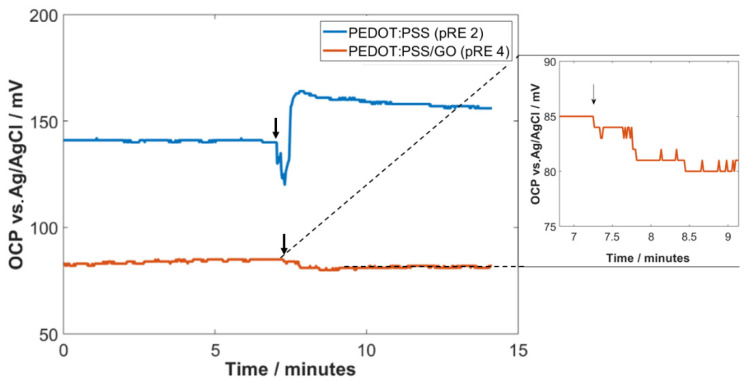
Impact of the addition of higher ionic strength droplets on the OCP of pRE 2 and pRE 4. The black arrows indicate the addition of high ionic strength solution.

To investigate the impact of changes in ion strength on the OPC of a PEDOT:PSS (pRE 2) and a PEDOT:PSS/GO electrode (pRE 4), the OCP measurements were first performed in 1 mM phosphate buffer (pH 7). During the recording of the OCPs, a phosphate buffer with higher ionic strength (100 mM, pH 7) was added to increase the ionic strength of the electrolyte. As shown in Figure 4, the OCP of the PEDOT:PSS electrode (pRE 2) showed a significant response to the addition of the high-concentration buffer. The OCP exhibited a highly unstable potential for almost 1 min after adding the 100 mM buffer and showed an overall potential change of approximately 25 mV. In comparison, the OCP of the GO-coated electrode (pRE 4) showed only a shallow change in its OCP of 1–2 mV.

The PEDOT:PSS/GO pRE 4 exhibited a highly stable OCP with a shallow drifting rate of 7 mV over 3 h. Compared to the pRE 2, the electrode potential does not need a specific time to become stable and exhibited a much lower drifting. This stable OCP behavior makes the electrode highly suitable for integrated FET biosensing applications. Furthermore, we could prove that the GO layer on top of the PEDOT:PSS thin film hinders the incorporation of ions into the polymer thin film. Therefore, the electrode potential is not dependent on the ion concentration of the surrounding electrolyte. This feature is of high importance when it comes to diagnostics with clinical samples because the ionic strength of these samples can differ from each other.

### 3.2. Sensing Performance

**Figure 5:**
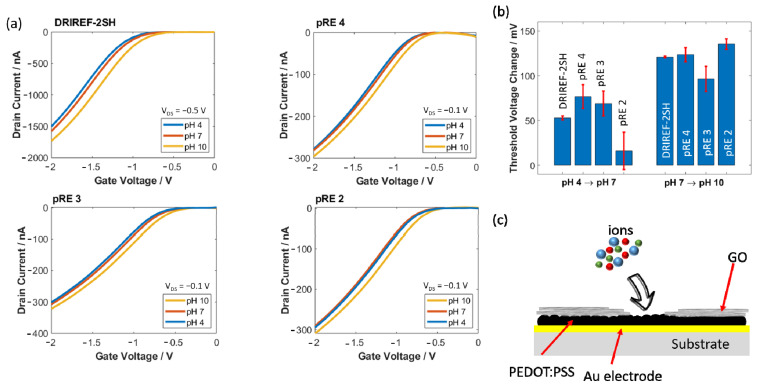
Transfer characteristics at different pH of a SiNW-FET gated with different gate electrodes (**a**). Threshold voltage change of a SiNW-FET due to changes in pH (**b**). Schematic illustration of the remaining cross-sensitivity and the reason for the relatively high standard deviation of our pRE (**c**).

To evaluate the ability of PEDOT:PSS-based electrodes as solid-state pREs, pH measurements were performed with SiNW-FETs, using the coated electrodes as the gate electrode. The transfer characteristics of the devices were measured with three different pH solutions (pH 4, pH 7, and pH 10) with different ionic strengths. The measurements were repeated four times per pH value (see Figure 5a). Besides using the PEDOT:PSS-based electrode, the pH sensitivity of the SiNW was also characterized using a commercial electrochemical Ag/AgCl electrode as a comparison. The transfer characteristics were obtained by sweeping the gate-source voltage from 0 V to −2 V at a constant drain-source voltage of −0.1 V or −0.5 V. Figure 5b shows the resulting threshold voltage change due to changes in pH using different kinds of pREs. All electrodes exhibited a larger threshold voltage change due to changing the pH from pH 7 to pH 10 compared to changing the pH from pH 4 to pH 7. Non-linear behavior of the pH response was observed due to the non-functionalized SiO_2_ surface of the gate oxide layer [45]. Here, the SiNW-FETs gated with our PEDOT:PSS/GO (pRE 4) exhibited only a slightly higher threshold voltage change compared to the devices gated with an electrochemical Ag/AgCl RE. A thinner PEDOT:PSS electrode coated with GO (pRE 3) led to lower threshold voltage changes, while the device showed the highest pH response using pRE 2. Changing the electrolyte from pH 4 to pH 7 resulted in a tiny and unpredictable change in threshold voltage when pRE 2 was used. Due to the significant difference in ionic strength between these two electrolytes, the large variation can be attributed to the remaining cross-sensitivity to ions. In addition, the threshold voltage change due to changes in pH may be superimposed with the remaining ion sensitivity of the pREs. The SiNW-FET exhibited a larger change in threshold voltage when using both PEDOT:PSS/GO electrodes compared to an Ag/AgCl RE. Overall, the SiNW-FET showed much higher reliability when an Ag/AgCl electrode was used compared to all other electrodes. A remaining cross-sensitivity to ions can explain the higher standard deviation of the polymeric pREs. As shown in Figure 1d, the GO film on top of the PEDOT:PSS had some micro-scale holes, which may allow ion diffusion into the PEDOT:PSS layer (illustrated in Figure 5c). In addition, pH measurements for pRE 1 are shown in Figure S10. The SiNW-FET exhibited a clear signal change due to changes in pH (95 mV due to changing the pH from 7 to 10). However, due to the lack of OCP stability, drifting during real-time measurements is expected. To verify this hypothesis, real-time measurements were performed using pRE 1 and pRE 4. As shown in Figure S11, a SiNW-FET gated with pRE 1 exhibits an unstable drain current over time, while a SiNW-FET gated with pRE 4 exhibits a stable drain current after a short drift.

The transfer characteristic of the SiNW-FETs was measured using two phosphate buffer solutions with the same pH (pH 7) and different ionic strengths (1 mM and 100 mM) to evaluate the ionic strength cross-sensitivity of the electrodes. The measurements were carried out for pRE 2 and pRE 4. The change in threshold voltage due to changes in ionic strength for the two electrodes is shown in Figure S12. It can be seen that the gating with a PEDOT:PSS electrode (pRE 2) resulted in a significant threshold shift of approximately 130 mV due to changing the ionic strength from 1 mM to 100 mM. In comparison, a SiNW-FET gated with a GO-coated PEDOT:PSS electrode (pRE 4) exhibited a lower threshold voltage shift of around 50 mV. These results match the above-mentioned OCP measurements. For both results, slight differences in pH need to be taken into account because the pH value of both solutions differed by around 0.2 pH. The remaining shift in threshold voltage identifies a remaining ion sensitivity of the GO-coated pRE. Here, the ion concentration differs by a factor of 100. The difference in ionic strength of the pH solutions used for the measurements shown in Figure 5 was less than a factor of 10. Therefore, the change in threshold voltage (compare Figure 5b) is mainly based on changes in pH with a minor but significant impact of the ion concentration of the electrolyte and a possible OCP drift of the pRE (as shown by the high standard deviation).

**Figure 6:**
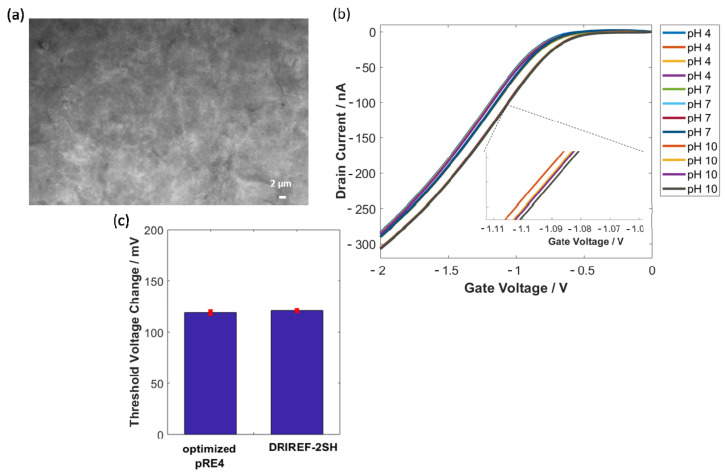
SEM image of the optimized GO coating (**a**). Multiple transfer characteristic measurements at different pH (**b**). Comparison in threshold voltage change due to changing the pH from 7 to 10 for an optimized pRE4 and a commercial Ag/AgCl RE (**c**).

A variation between single measurements using the presented pREs was observed, which may limit the use of pREs for sensing applications. This variation can be attributed to micro-scale holes inside the GO film and different immersion times into the electrolyte solution. To overcome the variability between different measurements under the same conditions, the GO coating of pRE 4 was further improved. Here, multiple small droplets were drop-casted on top of the PEDOT:PSS electrode to achieve a higher GO coverage. As shown in Figure 6a, the optimized drop-casting of the GO film results in a dense film without micro-scale holes in comparison to the non-optimized drop-casting (Figure 1d). A SiNW-FET gated with the optimized pRE 4 exhibits a distinguishable change in threshold voltage against electrolyte pH with an extremely reduced standard deviation (see Figure 6b and the inset). Changing the pH from 4 to 7 resulted in a threshold voltage change of 52 ± 3 mV (Ag/AgCl 53 ± 2 mV), and a change in pH from 7 to 10 resulted in a threshold voltage change of 119 ± 2 mV (Ag/AgCl 121 ± 1 mV) (see Figure 6c). These results show that the optimization in the GO film quality has great potential for overcoming the existing limitations of metallic and polymeric-based pREs (e.g., cross-sensitivity to the ionic strength of the electrolyte). Furthermore, due to the comparison of Figure 1d (non-optimized GO coating) and Figure 6a (optimized GO coating) it can be argued that micro-scale holes inside the GO film are the main reason behind the high standard deviation of pH measurements using a non-optimized GO-coated pRE.

Finally, we compared the potential drifting of our pRE 4 with several pRE approaches presented in the state-of-the-art literature. The comparison is shown in Table 3. Duarte-Cuevara et al. reported pREs based on metal electrodes coated with polypyrrole (PPy) [30]. Here, they have shown that the drifting of electrode potential can be reduced from 23.2 mV/h (only Pt) to 0.75 mV/h due to the coating of a Pt electrode with PPy. According to their finding, the metal has a huge impact on the stability of polymeric pRE. Using Au as an electrode material resulted in a ~2.9 times higher drift compared to a Pt electrode (both coated with PPy). Furthermore, they investigated the drifting of an electrochemical Ag/AgCl reference electrode that was found to be 0.6 mV/h. In comparison, our pRE approach (pRE 4) exhibited a drifting of 0.65 mV/h, which is comparable with the drifting of an Ag/AgCl RE [30]. Furthermore, we compared the achieved pH sensitivity using pRE 4 with other reported pRE concepts. Here, our findings show that the use of a GO-coated PEDOT:PSS electrode exhibited a similar pH sensitivity compared to an ISFET (with hafnium oxide dielectric) gated with a Pt electrode coated with PPy. However, it is noteworthy that the pH sensitivity of different devices should be compared carefully. For instance, the type and quality of the gate oxide are important for the pH sensitivity of such a device [46]. Additional data and information about the possibility of on-chip integration can be found in Table 3 to compare different pRE approaches. The achieved pH sensitivity of 39.7 mV/pH is comparable to the former results of our group [47].

## 4. Conclusions and Outlook

We have demonstrated that the coating of PEDOT:PSS electrodes with GO resulted in a much better pRE performance compared to bare PEDOT:PSS electrodes. The stability of the OCP was investigated by long-term measurements. The PEDOT:PSS/GO electrode exhibited a shallow drifting rate of 5 mV over the first 6 h and a slight drifting for the next 4 h and exhibited an overall constant electrode potential in most of the measurements. However, the pRE 4 configuration showed the potential of a low-drifting gate electrode with a minimal drift of 0.65 mV/h, which is comparable with state-of-the-art pREs. Transient OCP measurements were carried out by adding a 100 mM phosphate buffer (10% of the initial volume) to a 1 mM phosphate buffer of the same pH to evaluate the cross-sensitivity of the ionic strength of an electrolyte to the OCP of the electrode. A significant change in the OCP was observed for the PEDOT:PSS electrode, while the GO-coated PEDOT:PSS electrode showed a relatively stable behavior. The slight change in the OCP of GO-coated PEDOT:PSS electrodes can be attributed to micro-scale holes inside the GO film. Together with an electrochemical Ag/AgCl RE, the PEDOT:PSS and the GO-coated PEDOT:PSS electrodes were used with SiNW–FETs to characterize the pH sensitivity of the SiNW-FET for solutions with different ionic strength. The pH response of the SiNW-FET using the GO-coated PEDOT:PSS electrodes resulted in a similar pH response to the commercial electrochemical RE, while the SiNW-FET gated with a PEDOT:PSS electrode exhibited a partly unpredictable pH response. A change in the ionic strength led to a change in the threshold voltage of the SiNW-FET; however, the GO-coated PEDOT:PSS electrode showed an almost three times lower change compared to the PEDOT:PSS electrode. Furthermore, we have shown that the optimization in drop-casting of the GO resulted in a highly reliable and reproducible pH response. A SiNW-FET gated with a GO-coated PEDOT:PSS electrode exhibited the same pH response as one gated with a commercial Ag/AgCl RE.

In conclusion, a combination of GO with PEDOT:PSS by initial electropolymerization of PEDOT:PSS on a metal electrode and a subsequent coating of the electrode with GO has improved the performance of the electrode by lowering drifting of the OPC and eliminating the interference of the ionic strength to the OCP, a crucial characteristic of an RE. We assume that the GO has a function to stop the diffusion of ions in the electrolyte to the PEDOT:PSS layer underneath while maintaining the insensitivity to the pH of the PEDOT:PSS layer. One major drawback of the presented pRE is the remaining, but much lower, drifting and cross-sensitivity to the ionic strength of an electrolyte solution. The optimization of the GO coating further reduced the cross-sensitivity to the ionic strength of the analyte. A SiNW-FET gated with an optimized GO coating exhibited a similar threshold voltage change (ΔV_th_ 119 ± 2 mV) due to changing the analyte pH from 7 to 10 as one gated with an Ag/AgCl RE (ΔV_th_ 121 ± 1 mV). In comparison to the non-optimized GO coating, much higher reliability could be achieved due to optimization of the GO coating. With the help of SEM images, we could show that an optimized GO coating resulted in a continuous film without micro holes. Therefore, it can be concluded that the quality of the GO coating is highly influencing the performance of the pRE. The work presented here establishes the great potential of combining polymeric electrodes with ion diffusion barriers. In future work, we plan to utilize more controllable processes (e.g., spin-coating) to further improve the fabrication of a reliable on-chip pRE. In addition to the coating of the ion diffusion barrier, the impact of the metal underneath the polymer can be investigated to further improve the pRE performance.

## Figures and Tables

**Table 1:** Nomenclature of the investigated pREs.

Electrode Name	Electrode Composition
pRE 1	Au electrode coated with GO
pRE 2	Au electrode coated with PEDOT:PSS (termination charge of 100 µC)
pRE 3	Au electrode coated with PEDOT:PSS (termination charge of 10 µC), additional GO coating
pRE 4	Au electrode coated with PEDOT:PSS (termination charge of 700 µC), additional GO coating

**Table 2:** OCP changes at different points of the long-term recording shown in Figure 3.

Electrode Name	1 min	10 min	1 h	5 h	10 h
pRE 1	8.4%	6%	−0.9%	29%	39%
pRE 2	−0.4%	−2.8%	−12.6%	−30%	−48%
pRE 3	−15%	−21%	8.2%	2%	4%
pRE 4	−0.9%	−0.9%	−2.86%	−6.9%	2.7%

**Table 3:** Comparison of different pRE approaches in terms of drifting and achieved pH sensitivity of ISFETs gated with pREs.

pRE Concept	OPC Drift	V_th_ Change of pRE Gated ISFETs	Possibility of On-Chip Integration	Refs.
Ag/AgCl reference elelctrode	0.6 mV/h	54.9 mV/pH (hafnium oxide)	yes	[30]
Pt	23.2 mV/h	5.4 mV/pH (hafnium oxide)	yes	[30]
Pt + PPy	0.75 mV/h	44.2 mV/pH (hafnium oxide)	yes	[30]
Au + PPy	2.17 mV/h	-	yes	[30]
Palladium + PPy	0.92 mV/h	-	yes	[30]
Inkjet-printed pRE	4.16 mV/h	-	yes	[48]
Activated Carbon	0.8 mV/day	-	no	[49]
Ag/AgCl screen-printed	0.2 mV/h	-	yes	[50]
Ag/AgCl	0.2 mV/h	-	yes	[51]
PEDOT:PSS/GO	0.65 mV/h	39.7 mV/pH (silicon oxide)	yes	This work

## Data Availability

All relevant data generated or analyzed during this study are included in this published article and its supplementary information files.

## Supplementary Materials

The following supporting information can be downloaded at: https://www.mdpi.com/article/10.3390/s22082999/s1, Figure S1: V_th_ extraction method; Figure S2: Additional EIS data of PEDOT:PSS coated electrodes; Figure S3: Impact of deposition speed on the PEDOT:PSS film quality; Figure S4: 3D printed fluidic chambers; Figure S5: Inset of OCP measurements; Figure S6: Reproducibility of the OCP; Figure S7: Long-term OCP measurement; Figure S8: Low-drift OCP measurement; Figure S9: Additional OCP measurements of a PEDOT:PSS electrode; Figure S10: pH sensitivity of an SiNW-FET gated with pRE 1; Figure S11: Drain current stability using differen pREs; Figure S12: threshold voltage change due to changes in ionic strength.

## References

[1] Bergveld P. (1970). Development of an Ion-Sensitive Solid-State Device for Neurophysiological Measurements. IEEE Trans. Biomed. Eng..

[2] Pachauri V., Ingebrandt S. (2016). Biologically sensitive field-effect transistors: From ISFETs to NanoFETs. Essays Biochem..

[3] Ohno Y., Maehashi K., Matsumoto K. (2010). Label-free biosensors based on aptamer-modified graphene field-effect transistors. J. Am. Chem. Soc..

[4] Lu X.L., Munief W.M., Heib F., Schmitt M., Britz A., Grandthyl S., Muller F., Neurohr J.U., Jacobs K., Benia H.M. (2018). Front-End-of-Line Integration of Graphene Oxide for Graphene-Based Electrical Platforms. Adv. Mater. Technol..

[5] Figueroa-Miranda G., Liang Y., Suranglikar M., Stadler M., Samane N., Tintelott M., Lo Y., Tanner J.A., Vu X.T., Knoch J. (2022). Delineating charge and capacitance transduction in system-integrated graphene-based BioFETs used as aptasensors for malaria detection. Biosens. Bioelectron..

[6] Balasubramanian K., Burghard M. (2006). Biosensors based on carbon nanotubes. Anal. Bioanal. Chem..

[7] Torsi L., Magliulo M., Manoli K., Palazzo G.J.C.S.R. (2013). Organic field-effect transistor sensors: A tutorial review. Chem. Soc. Rev..

[8] Pachauri V., Vlandas A., Kern K., Balasubramanian K.J.S. (2010). Site-Specific Self-Assembled Liquid-Gated ZnO Nanowire Transistors for Sensing Applications. Small.

[9] Kaisti M. (2017). Detection principles of biological and chemical FET sensors. Biosens Bioelectron.

[10] Simonis A., Dawgul M., Luth H., Schoning M.J. (2005). Miniaturised reference electrodes for field-effect sensors compatible to silicon chip technology. Electrochim. Acta.

[11] Yee S., Jin H., Lam L.K.C. (1988). Miniature liquid junction reference electrode with micromachined silicon cavity. Sens. Actuators.

[12] Tintelott M., Pachauri V., Ingebrandt S., Vu X.T. (2021). Process variability in top-down fabrication of silicon nanowire-based biosensor arrays. Sensors.

[13] Hu Q., Chen S., Solomon P., Zhang Z. (2021). Ion sensing with single charge resolution using sub–10-nm electrical double layer–gated silicon nanowire transistors. Sci. Adv..

[14] Bergveld P. (2003). Thirty years of ISFETOLOGY—What happened in the past 30 years and what may happen in the next 30 years. Sens. Actuators B Chem..

[15] Lewenstam A., Inzelt G., Lewenstam A., Scholz F. (2013). Handbook of Reference Electrodes.

[16] Yang H., Kang S.K., Choi C.A., Kim H., Shin D.-H., Kim Y.S., Kim Y.T. (2004). An iridium oxide reference electrode for use in microfabricated biosensors and biochips. Lab Chip.

[17] Shinwari M.W., Zhitomirsky D., Deen I.A., Selvaganapathy P.R., Deen M.J., Landheer D. (2010). Microfabricated Reference Electrodes and their Biosensing Applications. Sensors.

[18] Petsagkourakis I., Kim N., Tybrandt K., Zozoulenko I., Crispin X. (2019). Poly(3,4-ethylenedioxythiophene): Chemical Synthesis, Transport Properties, and Thermoelectric Devices. Adv. Electron. Mater..

[19] Park H.-S., Ko S.-J., Park J.-S., Kim J.Y., Song H.-K. (2013). Redox-active charge carriers of conducting polymers as a tuner of conductivity and its potential window. Sci. Rep..

[20] Hempel F., Law J.K.Y., Nguyen T.C., Munief W., Lu X.L., Pachauri V., Susloparova A., Vu X.T., Ingebrandt S. (2017). PEDOT:PSS organic electrochemical transistor arrays for extracellular electrophysiological sensing of cardiac cells. Biosens. Bioelectron..

[21] Reineke S., Thomschke M., Lüssem B., Leo K. (2013). White organic light-emitting diodes: Status and perspective. Rev. Mod. Phys..

[22] Keene S.T., Lubrano C., Kazemzadeh S., Melianas A., Tuchman Y., Polino G., Scognamiglio P., Cinà L., Salleo A., van de Burgt Y. (2020). A biohybrid synapse with neurotransmitter-mediated plasticity. Nat. Mater..

[23] Schander A., Stemmann H., Tolstosheeva E., Roese R., Biefeld V., Kempen L., Kreiter A., Lang W. (2016). Design and fabrication of novel multi-channel floating neural probes for intracortical chronic recording. Sens. Actuators A Phys..

[24] Bobacka J. (1999). Potential Stability of All-Solid-State Ion-Selective Electrodes Using Conducting Polymers as Ion-to-Electron Transducers. Anal. Chem..

[25] Isaksson J., Kjäll P., Nilsson D., Robinson N., Berggren M., Richter-Dahlfors A. (2007). Electronic control of Ca^2+^ signalling in neuronal cells using an organic electronic ion pump. Nat. Mater..

[26] Berggren M., Malliaras G.G. (2019). How conducting polymer electrodes operate. Science.

[27] Paulsen B.D., Tybrandt K., Stavrinidou E., Rivnay J. (2019). Organic mixed ionic–electronic conductors. Nat. Mater..

[28] Heine V., Kremers T., Menzel N., Schnakenberg U., Elling L. (2021). Electrochemical Impedance Spectroscopy Biosensor Enabling Kinetic Monitoring of Fucosyltransferase Activity. ACS Sens..

[29] Kremers T., Tintelott M., Pachauri V., Vu X.T., Ingebrandt S., Schnakenberg U. (2021). Microelectrode Combinations of Gold and Polypyrrole Enable Highly Stable Two-electrode Electrochemical Impedance Spectroscopy Measurements under Turbulent Flow Conditions. Electroanalysis.

[30] Duarte-Guevara C., Swaminathan V.V., Burgess M., Reddy B., Salm E., Liu Y.S., Rodriguez-Lopez J., Bashir R. (2015). On-chip metal/polypyrrole quasi-reference electrodes for robust ISFET operation. Analyst.

[31] Han S., Polyravas A.G., Wustoni S., Inal S., Malliaras G.G. (2021). Integration of organic electrochemical transistors with implantable probes. Adv. Mater. Technol..

[32] Hempel F.W. (2019). Organic electrochemical transistors based on PEDOT: PSS for the sensing of cellular signals from confluent cell layers down to single cells. Mater. Sci..

[33] Leenaerts O., Partoens B., Peeters F. (2008). Graphene: A perfect nanoballoon. Appl. Phys. Lett..

[34] Bong J.H., Yoon S.J., Yoon A., Hwang W.S., Cho B.J. (2015). Ultrathin graphene and graphene oxide layers as a diffusion barrier for advanced Cu metallization. Appl. Phys. Lett..

[35] Yoo B.M., Shin H.J., Yoon H.W., Park H.B. (2014). Graphene and graphene oxide and their uses in barrier polymers. J. Appl. Polym. Sci..

[36] Sung S.J., Park J., Cho Y.S., Gihm S.H., Yang S.J., Park C.R. (2019). Enhanced gas barrier property of stacking-controlled reduced graphene oxide films for encapsulation of polymer solar cells. Carbon.

[37] Ren W., Cheng H.-M. (2014). The global growth of graphene. Nat. Nanotechnol..

[38] Yamaguchi H., Granstrom J., Nie W., Sojoudi H., Fujita T., Voiry D., Chen M., Gupta G., Mohite A.D., Graham S. (2014). Reduced graphene oxide thin films as ultrabarriers for organic electronics. Adv. Energy Mater..

[39] Lu X. (2018). Reduced Graphene Oxide Biosensors for Prostate Cancer Biomarker Detection. Ph.D. Thesis.

[40] Vu X., GhoshMoulick R., Eschermann J., Stockmann R., Offenhäusser A., Ingebrandt S. (2010). Fabrication and application of silicon nanowire transistor arrays for biomolecular detection. Sens. Actuators B Chem..

[41] Schnakenberg U., Benecke W., Lange P. TMAHW etchants for silicon micromachining. Proceedings of the TRANSDUCERS’91: 1991 International Conference on Solid-State Sensors and Actuators. Digest of Technical Papers.

[42] Klos J., Sun B., Beyer J., Kindel S., Hellmich L., Knoch J., Schreiber L. (2019). Spin Qubits Confined to a Silicon Nano-Ridge. Appl. Sci..

[43] Tintelott M., Ingebrandt S., Pachauri V., Vu X.T. Lab-on-a-chip based silicon nanowire sensor system for the precise study of chemical reaction-diffusion networks. Proceedings of the MikroSystemTechnik Congress 2021.

[44] Lazar J., Schnelting C., Slavcheva E., Schnakenberg U. (2016). Hampering of the stability of gold electrodes by ferri-/ferrocyanide redox couple electrolytes during electrochemical impedance spectroscopy. Anal. Chem..

[45] Cui Y., Wei Q., Park H., Lieber C.M. (2001). Nanowire nanosensors for highly sensitive and selective detection of biological and chemical species. Science.

[46] Fu W., Nef C., Knopfmacher O., Tarasov A., Weiss M., Calame M., Schönenberger C. (2011). Graphene transistors are insensitive to pH changes in solution. Nano Lett..

[47] Vu X.T., Eschermann J.F., Stockmann R., GhoshMoulick R., Offenhäusser A., Ingebrandt S. (2009). Top-down processed silicon nanowire transistor arrays for biosensing. Phys. Status Solidi A.

[48] Papamatthaiou S., Zupancic U., Kalha C., Regoutz A., Estrela P., Moschou D. (2020). Ultra stable, inkjet-printed pseudo reference electrodes for lab-on-chip integrated electrochemical biosensors. Sci. Rep..

[49] Abbas Y., Olthuis W., van den Berg A. (2015). Activated carbon as a pseudo-reference electrode for electrochemical measurement inside concrete. Constr. Build. Mater..

[50] Ying K.S., Heng L.Y., Hassan N.I., Hasbullah S.A. (2020). A New and All-Solid-State Potentiometric Aluminium Ion Sensor for Water Analysis. Sensors.

[51] Tymecki Ł., Zwierkowska E., Koncki R. (2004). Screen-printed reference electrodes for potentiometric measurements. Anal. Chim. Acta.

