# Supplementary material for: Realization of a PEDOT:PSS/Graphene Oxide On-Chip Pseudo-Reference Electrode for Integrated ISFETs
Source: Sensors (Basel). 2022 Apr 14;22(8):2999. doi: 10.3390/s22082999 (PMC9032565; doi:10.3390/s22082999)
Supplement: Supplementary file 1 [file sensors-22-02999-s001.zip › sensors-1549163-supplementary.pdf]

Supporting Information

Realization of a PEDOT:PSS/Graphene Oxide On-Chip Pseudo Reference Electrode for Integrated ISFETs

Marcel Tintelott, Tom Kremers, Sven Ingebrandt, Vivek Pachauri and Xuan Thang Vu *

Institute of Materials in Electrical Engineering 1, RWTH Aachen University, Sommerfeldstr. 24, 52074 Aachen, Germany

* Correspondence: vu@iwe1.rwth-aachen.de; Tel.: +49-241-80-27816

Keywords: PEDOT:PSS, 2D materials, biosensor, stability, gate electrode, diffusion barrier

Materials and Methods

1: Extraction for the threshold voltage

Due to the variety of extraction methods for the threshold voltage of field-effect transistors, the used method is briefly described in the following. We utilized the transconductance extrapolation method in the linear region. As shown in figure S-1 the gate voltage axis intercept of the linearly extrapolated $g_m - V_{gs}$ characteristics at its maximum slope corresponds to the threshold voltage.

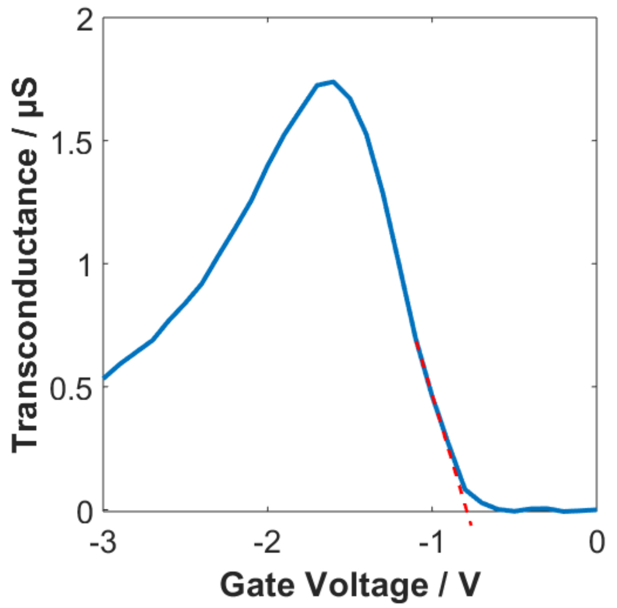

Figure S1. Threshold voltage extraction based on the transconductance extrapolation method.

Results

2: Further EIS spectra of PEDOT:PSS coated electrodes

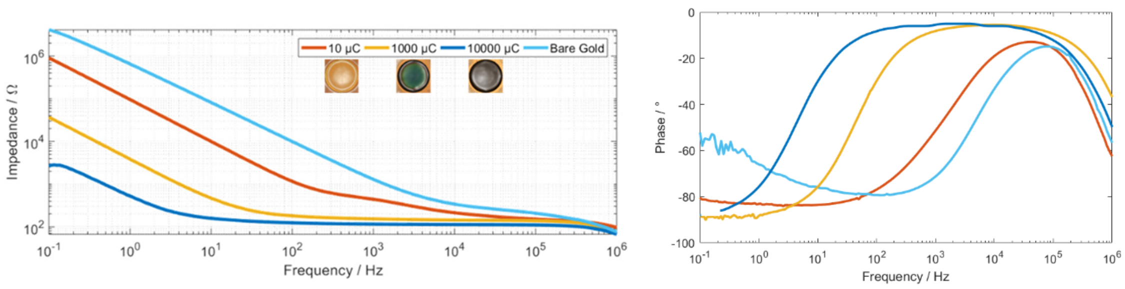

Figure S2. Bode plots of electrochemical impedance spectra (top) and phase (bottom) of PEDOT:PSS coated electrodes and a bare gold electrode. An increasing PEDOT:PSS layer thickness results in a lower electrode impedance.

3: Impact on deposition speed on the PEDOT:PSS film quality

The impact of deposition speed on the PEDOT:PSS film quality is shown in figure S-3.

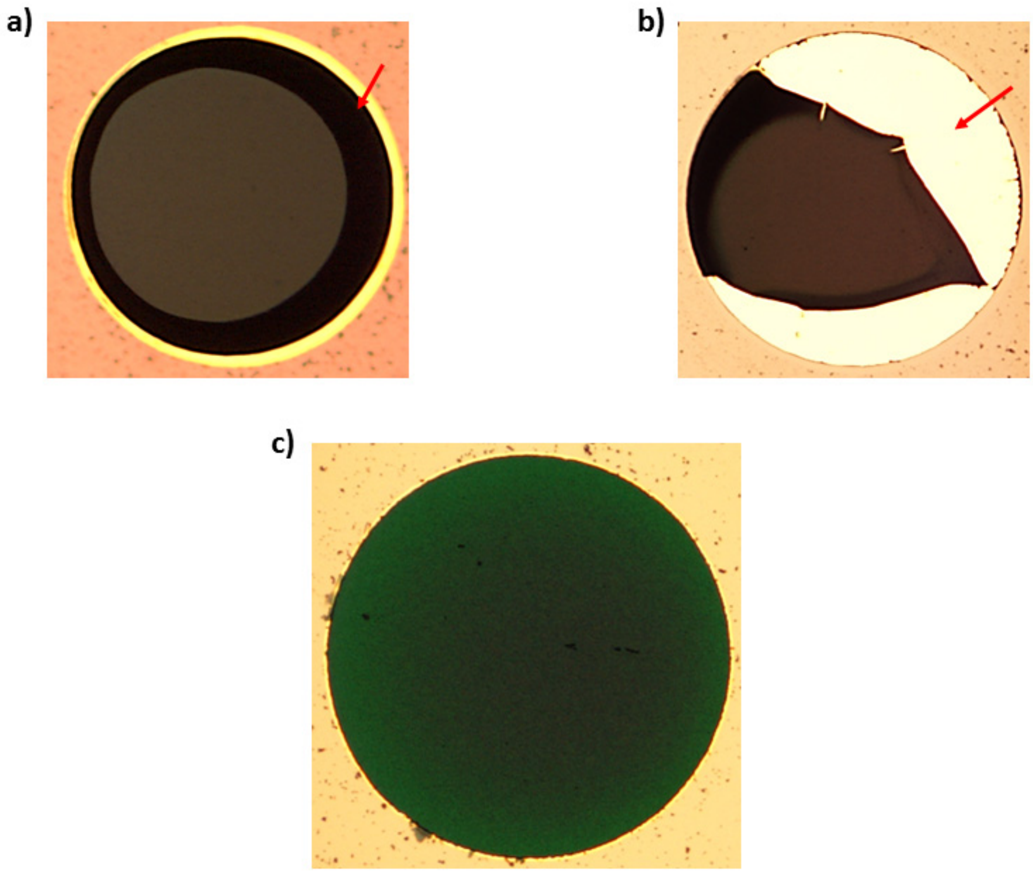

Figure S3. Fast deposition using a large counter electrode (1 cm x 1 cm): (a) start of delamination and (b) delaminated of a PEDOT:PSS film. (c) shows the result of a slow deposition using two equally sized electrodes.

4: 3D printed fluidic chambers for electropolymerization, EIS measurements, and OCP recordings

The 3D printed fluidic chambers have been fabricated to allow a highly controllable measurement scheme and to reduce the variation during electropolymerization. A fused deposition modeling (FDM) has been used for the printing of the two different fluidic chambers. We designed one chamber for electrical measurements which allows a user-friendly insertion of a small-scale Ag/AgCl reference electrode for OCP or EIS measurements (as seen in figure S-4 (left)). On the right side of figure S-4 a fluidic chamber for the electropolymerization process can be seen. It is noteworthy that the fluidic chambers exhibited a strong leakage of the electrolyte solutions. To ensure a long-term immersion into an electrolyte solution, the 3D printed fluidic chambers have been coated with a 3.5 µm thick parylene C layer.

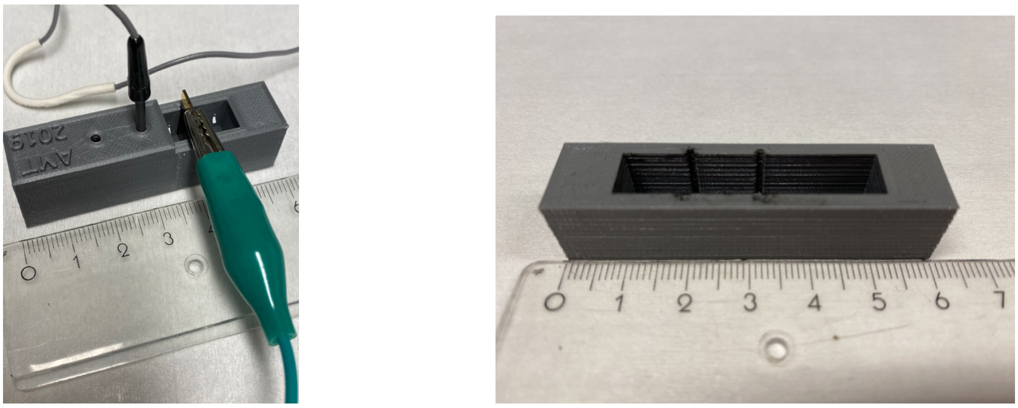

Figure S4. Images of the two fluidic chambers used for the electropolymerization process (right) and EIS and OCP measurements (left).

5: Inset of the measurement shown in figure 3

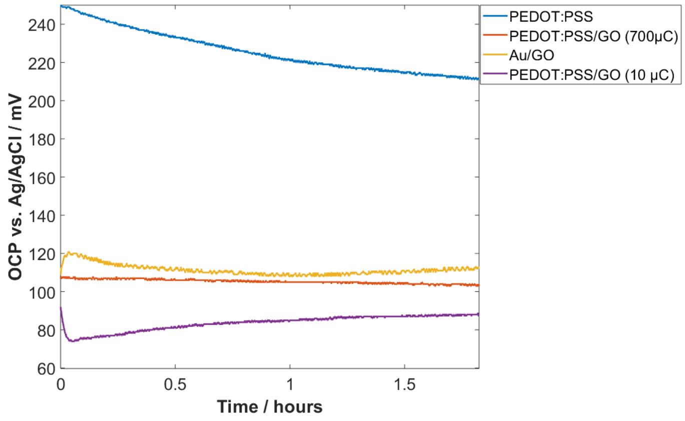

Figure S5. Inset of OCP measurements shown in the main text in figure 3.

6: Reproducibility of the OCP

To ensure that the stable OCP measurements were no single-shot measurements, multiple measurements have been performed. Figure S-5 (left) shows an example of two OCP recordings of our PEDOT:PSS/GO pRE for 20 minutes in 1 mM phosphate buffer (pH 7). The investigated electrodes exhibited a stable OCP during the whole recording period. Only a slight variation in the OCP of the two electrodes can be observed (figure S-5 (left)). Furthermore, a transient OCP measurement for 3 hours has been performed (figure S-5 (right)). Here, the PEDOT:PSS/GO electrode exhibits a drifting of around 7 mV.

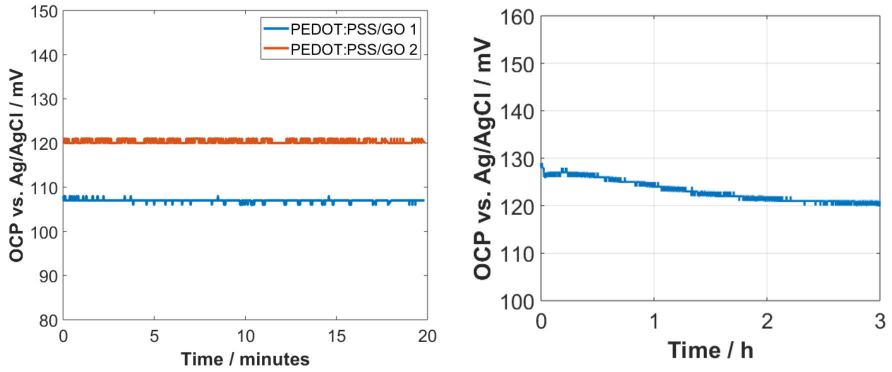

Figure S6. Transient OCP measurements of two PEDOT:PSS/GO pREs for 20 minutes (left). Long-term OCP measurement of an PEDOT:PSS/GO electrode (right).

7: Long-term OCP measurement of pRE 4

This section shows supporting long-term OCP measurements over 73 hours. The electrode (pRE 4) exhibited a stable potential for around 10 hours, while it showed a significant drifting after 10 hours.

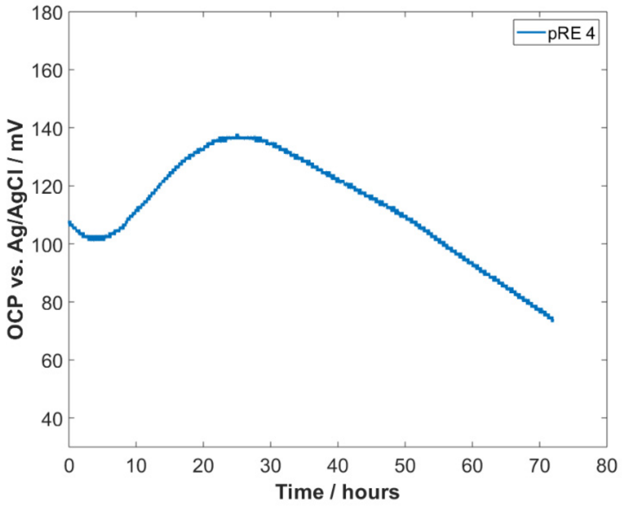

Figure S7. A 73 hours recording of the OCP of pRE 4.

8: Low-drifting OCP measurement of pRE 4

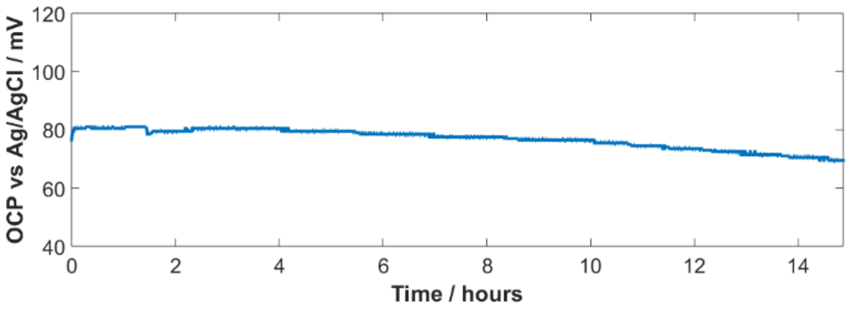

Figure S8. Low-drifting OCP measurement of a pRE 4 design. The electrode exhibited a drifting of 0.65 mV/h within the first 10 hours.

9: OCP measurement of a bare PEDOT:PSS electrode

In this section supporting OCP measurements of PEDOT:PSS electrodes are shown. As shown in the main text, these electrodes also exhibited a strong drifting behaviour.

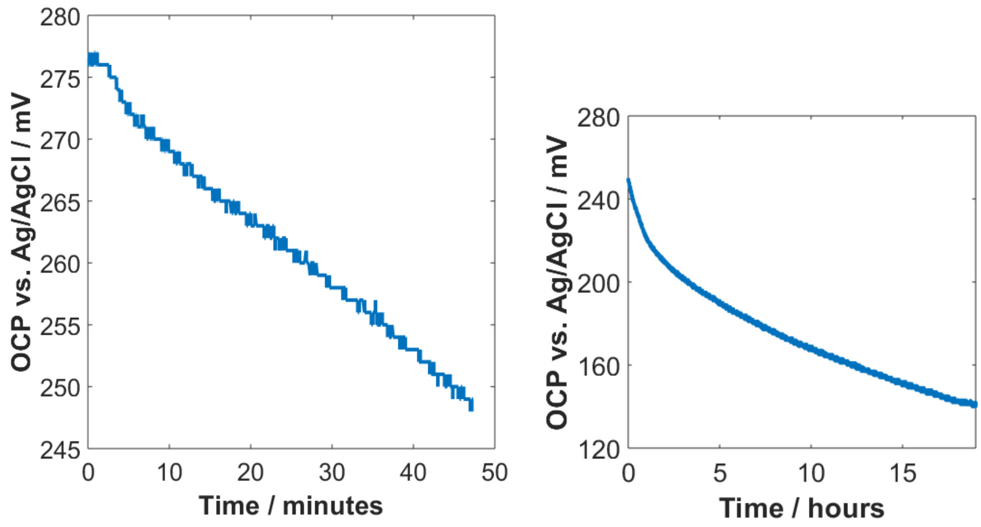

Figure S9. Transient OCP measurements of two PEDOT:PSS (100 μC termination charge) electrode. The OCP exhibits a continuous drift for the whole measurement for both short-term (left) and long-term (right) measurements.

10: pH sensitivity of an SiNW-FET gated with pRE 1

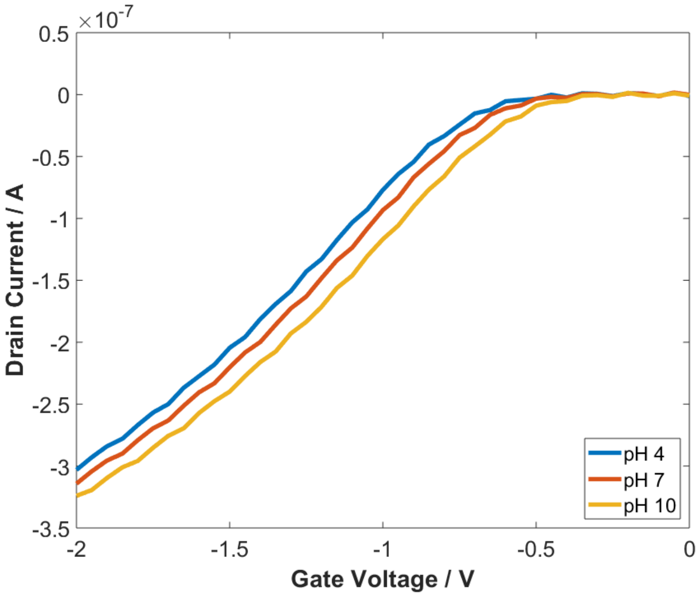

Figure S10. Transfer characteristic of a SiNW-FET gated with pRE 1 using different pH solutions. The system exhibited a threshold voltage change of 68 mV from pH 4 to pH 7 and a change of 95 mV from pH 7 to pH 10.

11: Drain current stability of SiNW-FETs gated with pRE1 and pRE4

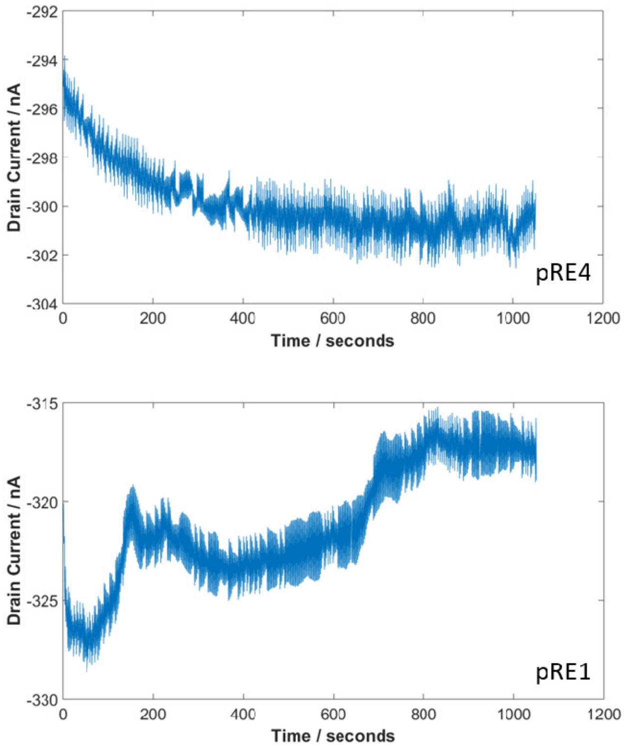

Figure S11. Drain current recording of a SiNW-FET gated with pRE1 (bottom) and pRE4 (top) in PBS (pH 7.4).

12: Drain current stability of SiNW-FETs gated with pRE1 and pRE4

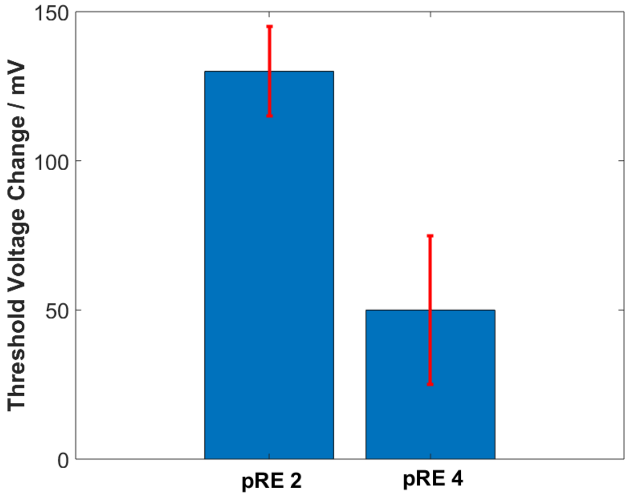

Figure S12. Threshold voltage change of a SiNW-FET gated with a bare PEDOT:PSS and a PEDOT:PSS/GO pRE due to changes in ionic strength from 1 mM to 100 mM.
